# Global environmental plastic dispersal under OECD policy scenarios toward 2060

**DOI:** 10.1126/sciadv.adu2396

**Published:** 2025-04-16

**Authors:** Jeroen E. Sonke, Alkuin Koenig, Théo Segur, Nadiia Yakovenko

**Affiliations:** ^1^ Géosciences Environnement Toulouse, CNRS/IRD/Université de Toulouse, 31400 Toulouse, France.; ^2^ Institut des Géosciences de l’Environnement, Université Grenoble Alpes, CNRS, IRD, Grenoble INP, 38400 Grenoble, France.; ^3^ Helmholtz-Zentrum Hereon, Institute of Coastal Research, 21502 Geesthacht, Germany.

## Abstract

Recent studies and OECD (Organization for Economic Cooperation and Development) reports provide roadmaps to reduce dispersal of mismanaged plastic waste to aquatic environments. Here, we use a coupled land-ocean-atmosphere model to simulate global plastic and microplastic dispersal for different OECD policy scenarios toward 2060. We establish a global plastic budget for the year 2015, with revised estimates of the total marine plastic pool of 263 teragrams (Tg, million tons), and land to sea plastic transport of 14 Tg per year, implying four to nine times larger leakage than OECD estimates. Model simulation of two ambitious policy scenarios show a peak in land to sea transport of total plastics of 23 Tg per year around 2045 and a decrease thereafter. Environmental concentrations of small microplastics remain high after 2060 due to continuous fragmentation of legacy mismanaged waste on land and indicate the need for remediation of legacy terrestrial plastic waste in policy instruments.

## Teaser

Global plastic pollution budget finds most historical plastic waste on land, indicating need for remediation policy instruments.

## INTRODUCTION

Plastics occupy a central role in the global economy yet cause substantial damage to ecosystems and human health (*1*, *2*). From 1950 to 2023 humans have produced 10,000 teragrams (Tg, 10^12^ grams, millions of metric tons) of fossil fuel–based polymers, an amount growing at a rate of 3% per year (*3*). Life cycle analysis has kept track of the fate of produced plastic polymers, in terms of usage (30%), waste management by incineration (10%), recycling (5%), landfilling (35%), and mismanagement (20%) (*3*, *4*). Landfilled and mismanaged, dumped plastic waste slowly fragments to microplastic at a rate of about 3% per year (*5*), adding to the substantial (14%) fraction of primary microplastics in waste (*6*). Both large plastic debris and microplastics disperse (also called “leaking”) by continental runoff and wind to pollute natural terrestrial and marine ecosystems (*7*) and have been documented across all continents and seas from mountain tops to ocean trenches (*8*, *9*). Understanding past and future fragmentation and dispersal of plastics and microplastics is critical to determine their health impact on wildlife and humans (*10*). Plastic debris entangles aquatic species, and during use, after disposal, and upon ingestion plastics release toxic additives that disrupt endocrine function and increase risk for disease or disorders (*2*). International efforts are underway to curb the impact of plastic pollution, and different environmental policy scenarios have been proposed to limit dispersal (*4*, *6*).

Integrated understanding of global plastics dispersal, however, has been challenging thus far, with efforts focused on either marine (*11*, *12*) or terrestrial environments, sometimes in interaction with the atmosphere (*13*–*15*), but rarely addressing coupled biogeochemical cycling of plastics in the Earth surface system (*16*). Recent studies on plastics and microplastics in the surface and deep ocean, in marine sediments, and in the atmosphere have provided critical information on plastics cycling and dispersal. The amount of buoyant plastics at the ocean surface, around 2 Tg (*12*, *17*), is just the tip of the marine plastics iceberg, with potentially up to 80 Tg of microplastics suspended in the deep ocean (*16*) and between 120- and 250-Tg deposited in marine sediments (*16*, *18*, *19*). The organization for economic cooperation and development (OECD) estimate of plastic waste dispersal to the marine environment is smaller, around 30 Tg (*4*), underlining the incomplete understanding of global environmental plastics leakage.

In its 2022 “*Global Plastics Outlook: Policy Scenarios to 2060*” report (*4*), the OECD outlined two environmental policy scenarios to reduce the environmental impacts of plastics: the Regional Action and Global Ambition. These policy scenarios contrast with the business as usual (BAU) base case that projects a tripling of plastic production and waste generation by 2060. The Regional Action scenario is based on improved circularity of plastics and reduced plastic pollution (leakage). Overall plastic production and waste generation decreases (by taxing use) by 18% compared to BAU, recycling increases to 40%, and the decrease in mismanaged waste and leakage is mostly driven by improvements in waste management in non-OECD countries. The Global Ambition scenario is based on rapidly improved circularity of plastics and reduced plastic leakage in all countries, including near-zero plastic pollution by 2060. Plastic production decreases by 33% compare to BAU, recycling increases to 60% globally, and almost all the increase in demand for plastics can be met by recycled secondary plastics. In this study, we integrate recent observations on environmental plastics and microplastics in a global plastic cycling box model, and use the model to simulate environmental plastics levels in water, soil, sediment and air under the OECD BAU, Regional Action and Global Ambition scenarios. We also evaluate the independent “system change scenario” (SCS) from Lau *et al.* (*6*), which, similar to Global Ambition, proposes forceful but realistic measures to reduce, substitute, recycle, and dispose of plastics. Compared to the OECD Global ambition scenario, the SCS has a similar increase in recycling rate of more than 50% by 2060 but has twice lower primary plastic production of 440 Tg year^−1^, half provided from recycled plastics. The policy instruments behind all four scenarios rely on taxation of plastic products, increased recycling and durability, sanitary landfill development, increased producer responsibility, and financial aid to developing countries (*4*).

## RESULTS AND DISCUSSION

### Global plastics budget and cycle

The GBM-Plastics v1.1 global box model (file S2) builds on earlier work (*16*) and simulates how plastic waste is dispersed through the terrestrial, marine, and atmospheric environments upon release and emission. Three broad size classes of plastic waste are included: macroplastic (P, >5 mm), large microplastic (LMP, >0.3 mm and <5 mm) and small microplastic (SMP; <0.3 mm, implicitly including nanoplastic). We also use MP to denote the sum of SMP and LMP because MP is often reported in observational studies. The box model does currently not consider regional variability and therefore uses globally averaged values for forcings (plastic production and waste generation statistics) and for recent environmental plastics observations on land, in air, and in oceans. In the model, P fragment to LMP and LMP fragment to SMP at a rate of 3 ± 1% per year, which is highly simplified yet in agreement with observed fragmentation rates for common mismanaged plastic waste (MMPW) objects including bottles, food containers, and agricultural mulch (*5*). A rate of 3% per year has also been found to satisfactorily explain the distribution of macro and microplastic in marine plastics dispersion models (*11*, *12*). In the box model, only SMP become airborne, emitted from both oceans and land. GBM-Plastics is a first order box model, meaning that a plastic flux, *F*, out of a reservoir is linearly proportional to the mass, *M*, of plastics in the reservoir, i.e., *F* = *k* × *M*. The mass transfer (rate) coefficients, *k*, are derived for each flux from published observations and estimates of *F* and *M* for the period 2006–2024, from observed degradation or deposition rates, or by fitting.

In GBM-plastics v1.1, we expanded the following features and parameterizations: Uncertainty analysis is improved with a full Monte Carlo approach for all main input parameters and forcings. Terrestrial discarded pools of P, LMP, and SMP are now subdivided into landfilled and MMPW pools, each receiving 50% of discarded waste from 1950 to 2015 and variable fractions beyond 2015 (*4*). We also integrated the substantial, 45 ± 14%, open burning of MMPW (*4*, *6*). Modeled plastics in sanitary landfills do not leak to their surrounding environment, including oceans, over the timescale considered in this study (1950–2100). Over longer timescales, the erosion of landfills may have to be considered (*16*). GBM-plastics v1.1 model forcings for 1950–2015 consist of plastic production and waste generation statistics from Geyer *et al.* (*3*), which were also adopted by the OECD and extended to 2019. The release of primary (intentionally manufactured) microplastic to the environment from 1950 to 2015 is scaled to the total MMPW leakage statistics. The fraction of annual MMPW that is microplastic is taken from Lau *et al.* (*6*) and is 14 ± 3% (1σ) and is held constant from 1950 to 2015. The release of secondary microplastic (both SMP and LMP that are produced by fragmentation) from 1950 to 2015 and to 2100 follows the first-order mass transfer relationships, meaning that secondary LMP generation from P is linearly proportional to the mass of P in a reservoir and that LMP dispersal is linearly proportional to the slowly accumulating LMP mass in the same reservoir. For further model details, see Materials and Methods.

**Fig. 1.**
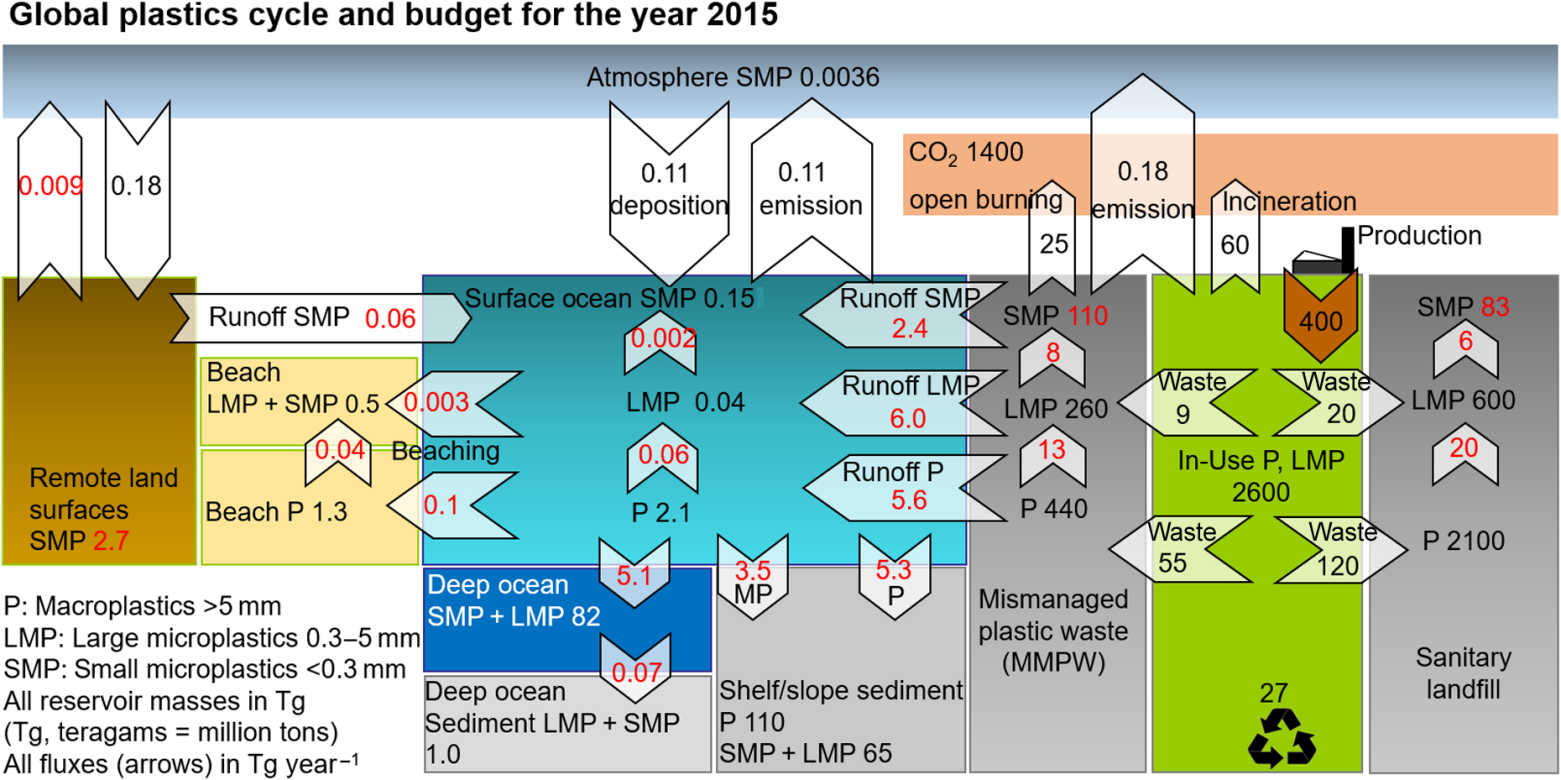
Global plastics budget and cycle for the year 2015. Reservoir sizes are shown in teragrams (Tg, million tons), and fluxes in Tg year^−1^ (arrows). Three plastics size classes are considered: P >5 mm, LMP from 0.3 to 5 mm, and SMP <0.3 mm that can become airborne. Plastic waste is impounded in sanitary landfills, incinerated, or discarded as MMPW, which is subject to open burning and dispersal to the marine environment. The remote land surface reservoir (soils, rock, glaciers, and deserts) covers 97% of land and is only affected by airborne SMP deposition, re-emission, and runoff. Numbers in black are based on production and waste statistics and on environmental P and MP observations (over the period 2006–2024). Numbers in red are estimated from the box model simulation. Uncertainties are summarized in table S1.

We update our best estimate of the global environmental plastics cycle for the year 2015 in Fig. 1 (see table S1 for uncertainties). Where possible, numbers (in black) are based on observations over the period ~2006–2024 or on published atmospheric modeling results fitted against observations (see Materials and Methods). Nevertheless, a certain number of plastic pools and fluxes (in red) are estimated from the GBM-plastics model and need empirical observations for further comparison. In the global plastics budget (Fig. 1 and Materials and Methods), we adopt a median marine SMP emission flux of 0.11 Tg year^−1^ [interquartile range (IQR): 0.08 to 2.3; Table 1) and complete it with a similar consensus estimate of land SMP emissions of 0.18 Tg year^−1^ (median, IQR: 0.16 to 0.44; Table 1). We also adopt a revised global atmospheric SMP burden of 0.0036 Tg (median, IQR 0.002 to 0.017; Table 1). We previously estimated a shelf sediment P pool of 51 Tg based on (*20*). A recent review compiled published microplastic and mesoplastic data in the range of 10 μm to 25 mm to estimate a global marine sediment pool of 170 Tg (>99% P; range of 25 to 900 Tg) (*18*). Another review estimates the global marine sediment P pool to be 7 Tg (range of 3 to 11 Tg) from remote operated vehicle studies and 255 Tg (range of 5 to 571 Tg) from bottom trawl studies (*19*). Based on these studies, we derive a best estimate marine shelf and slope sediment P pool of 110 Tg (median, IQR: 40 to 191 Tg; Table 2). Surface ocean buoyant P and LMP pools were previously estimated to be on the order of 0.23 and 0.04 Tg (*21*). The inclusion of larger plastic debris in the floating P inventory and the availability of more data have recently produced 10 times larger floating P estimates of 2.3 Tg (*17*) and 1.9 Tg (*12*) (Table 2). Table 3 summarizes surface mixed layer SMP observations with a median concentration of 8.5 μg m^−3^ (IQR: 1.5 to 44), corresponding to a global surface ocean SMP pool of 0.15 Tg (median, IQR: 0.03 to 0.79).

**Table 1.** Microplastic emission estimates from ocean and land. Published marine and land SMP emission flux estimates, and atmospheric SMP burden, reported as median and IQR values.

Study	Marine SMP emission fluxTg/year	Method	Marine data	Atmospheric data	Upper SMP size emittedμm	Atmospheric SMP burdenTg	Land SMP emission fluxTg/year
Brahney *et al.* (*13*)	8.6 (0–22)	Modeling (CAM)	(*37*)	(*13*)	70	0.0036	0.18
Evangeliou *et al.* (*14*)	8.9 ± 3.5	Modeling (Flexpart)	(*37*)	(*13*)	250		0.69
Yang *et al.* (*38*)	0.00077 (0.00003–0.0015)	Experiments	(*37*)		70		
Fu *et al.* (*15*)	0.12 (0.035–0.44)	Modeling (GEOS-Chem)	(*39*)	(*40*)	70	0.00051	0.13
Shaw *et al.* (*26*)	0.10 (0.02–7.4)	Experiments	(*12*)		500		
Sonke *et al.* (*16*)				(*8*, *41*)		0.031	
**Median, IQR (25th–75th)**	**0.11 (0.08–2.32)**					**0.0036 (0.002–0.017)**	**0.18 (0.16–0.44)**

**Table 2.** Plastic and microplastic mass in the marine system. Published marine P, LMP and SMP global pool estimates, in Tg, as median and IQR (25th and 75th percentiles) or mean ± SD. Some studies report the sum of LMP and SMP, indicated by MP here. ROV, remote-operated vehicle.

Study	Beach		Surface ocean			Deep ocean	Deep sediment	Shelf/slope sediment	Shelf/slope sediment	Total marine		
	P	MP	P	LMP	SMP	MP	MP	P	MP	MP	P	P + MP
Sonke *et al.* (*16*)	1.3	0.5	0.23	0.036	0.003	82	1.0	51	65	149	52	201
Martin *et al.* (*18*)								170				
Zhu *et al.* - ROV (*29*)								3.2				
Zhu *et al.* - Trawl (*29*)								255				
Kaandorp *et al.* (*12*)			1.9	0.051								
Eriksen *et al.* (*21*)				0.036								
Eriksen *et al.* (*17*)			2.3									
This study					0.15							
**Median/mean (Tg)**	**1.3**	**0.5**	**2.1**	**0.044**	**0.15**	**82**	**1.0**	**110**	**65**	**149**	**114**	**263**
SD			0.3	0.011		27						
IQR (25th)					0.02			39	21	56	40	96
IQR (75th)					0.32			191	78	207	197	404

**Table 3.** Surface ocean SMP concentrations. Published marine SMP (<300 μm) concentrations in the mixed layer (50 m) and estimated global pool, in Tg, as median and IQR (25th and 75th percentiles). N, north; S, south.

Study	Basin	Depth, m	SMP, μg m^−3^	SMP, Tg
Pabortsava and Lampitt (*42*)	N-S Atlantic	10–70 m	860	
Zhao *et al.* (*43*)	S-Atlantic	10–60 m	2.0	
Eo *et al.* (*44*)	Korean East Sea	15–58 m	120	
Poulain *et al.* (*36*)	N-Atlantic	0–0.2 m	0.016	
Ross *et al.* (*45*)	Arctic	5–21 m	2.7	
Kanhai *et al.* (*46*)	Arctic	8–100 m	0.010	
Tekman *et al.* (*47*)	Arctic	1–3 m	18	
Enders *et al.* (*35*)	N-Atlantic	3 m	15	
**Median**			8.5	0.15
IQR (25th)			1.5	0.03
IQR (75th)			44	0.79

Key properties of the 2015 global plastics dispersal budget and cycle are the following: The substantial mass of plastics, 263 Tg (median, IQR: 96 to 204) that has polluted the marine environment, represents 3% of the 8100 Tg of plastics produced since the year 1950. The subsurface oceanic MP (82 ± 27 Tg) and shelf sediment P and MP (175 Tg, IQR: 60 to 269) reservoirs are substantially larger than the beached P and MP (1.8 ± 1.4 Tg) and surface ocean floating P and MP pools (2.3 ± 0.3 Tg). This implies that surface ocean plastics are just the tip of the marine plastics iceberg. The large subsurface ocean and sediment plastic pools also solve the missing marine plastics paradox and constrain the substantial land to sea inputs of P (5.6 ± 2.2 Tg year^−1^), LMP (6.0 ± 1.9 Tg year^−1^), and SMP (2.4 ± 0.9 Tg year^−1^), totaling 13.9 ± 3.9 T year^−1^ in 2015, which are required to explain the 263 Tg of plastics that have accumulated in the marine environment since 1950. Despite the large amounts of marine plastic pollution, most MMPW resides on land, in dumps, and discarded on land (discounted for open burning of MMPW), where it drives dispersion to air and oceans: MMPW on land amounts to 440 ± 130 Tg of P, 260 ± 53 Tg of LMP, and 110 ± 35 Tg of SMP in 2015. Managed plastic waste also resides on land, in the form of the large mass of plastics discarded to landfills, 2100 ± 260 Tg of P, 600 ± 120 Tg LMP, and 83 ± 27 Tg SMP, where it is immobilized temporarily, but not on millennial timescales (*16*).

### OECD and SCS policy scenarios

**Fig. 2.**
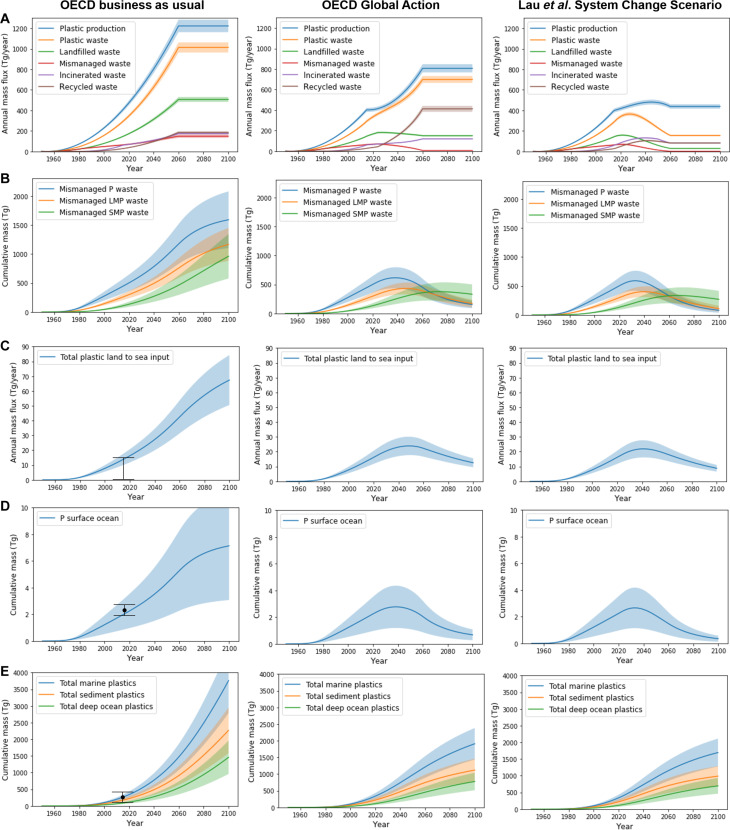
Model simulations of global plastics dispersal. Four plastics production and waste management scenarios (columns) are simulated from 2016 to 2100: OECD BAU, OECD Regional Action (not shown here; see fig. S1), OECD Global Action, and the System Change Scenario (SCS) from (*6*). From 1950 to 2015 all scenarios use production and waste management statistics from (*3*). From 2060 to 2100, the statistics are fixed to the policy scenario end-point values at 2060. For each scenario, the panels show (**A**) annual plastic production and waste generation, (**B**) cumulative MMPW, (**C**) annual land to sea plastic flux, (**D**) cumulative floating marine macroplastic, and (**E**) cumulative plastic mass in the marine system. Observed land to sea plastic transport, surface ocean macroplastic, P, and total marine plastics pool are shown in (C) to (E) for BAU (black circles and error bars, mean ± SD, or range). SMP, <300 μm; LMP, 300 to 5000 μm; P, >5000 μm.

**Fig. 3.**
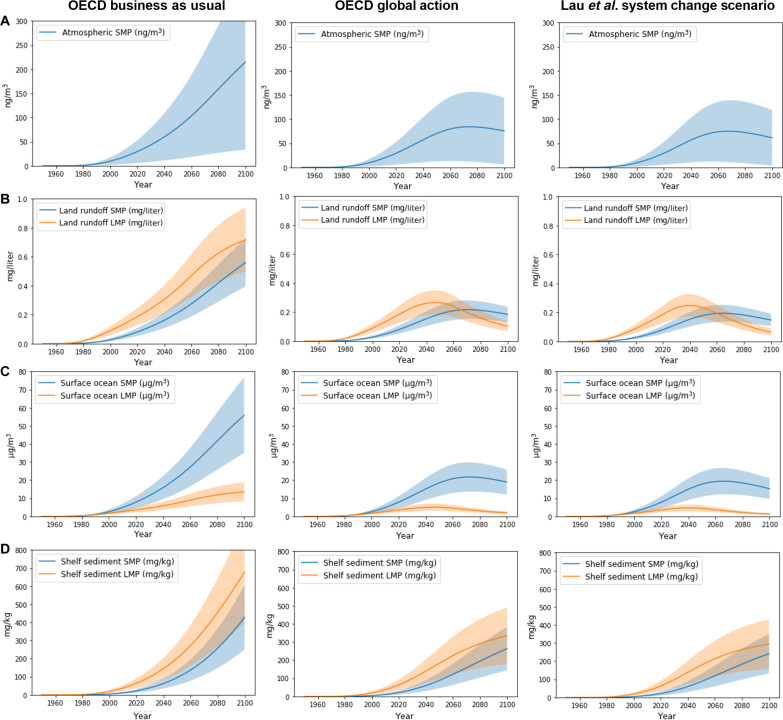
Model simulations of microplastic concentrations in air, terrestrial runoff, surface ocean and sediment. GBM-Plastics simulations of four plastics production and waste management scenarios (columns) from 2016 to 2100: OECD BAU, OECD Regional Action (not shown here; see fig. S2), OECD Global Action, and the SCS from (*6*). For each scenario, the panels show microplastic concentrations in (**A**) the atmosphere (troposphere), (**B**) land runoff to oceans, (**C**) surface ocean mixed layer (50 m), and (**D**) continental shelf sediment. SMP, <300 μm; LMP, 300 to 5000 μm.

Plastic production and waste management statistics at the global scale are summarized in Fig. 2A, fig. S1A, and file S1 for the four policy scenarios and include past and projected quantities of plastic waste that is incinerated, recycled, and discarded (landfilled and mismanaged). The model is then run from 1950 to 2100, with production and waste statistics as external forcing. From 2060 to 2100, the model forcings are held constant at the 2060 values to illustrate the long-term plastics cycling dynamics for the different scenarios toward the end of the 21st century. Simulation results for the OECD BAU and Global and the SCS policy scenarios are summarized in Figs. 2 and 3 for key metrics. Figure 2A illustrates the level of policy ambitiousness in terms of annual plastics production, reaching 1200 (BAU), 810 (Global) and 440 Tg year^−1^ (SCS) in 2060 compared to 400 Tg year^−1^ in 2015. We recall that total plastics production is the sum of virgin plastic production and recycled, secondary plastic production. Figure 2A and fig. S1A show waste management and end-of-life trajectories, projecting notably a phase out of MMPW by 2060 for the Global and SCS scenarios. Recycling by 2060 is progressively more ambitious from BAU (18%) to Regional (39%) to Global (59%) and SCS (53%) scenarios, while incineration stays around 20% in the three OECD scenarios and 32% in SCS. Figure 2B and fig. S3B track the large amounts of cumulative landfilled waste and terrestrial MMPW, showing that despite the stabilization (Regional) or even decrease (Global and SCS) in the total amount of mismanaged P waste, the quantities of mismanaged SMP waste keep increasing toward and beyond 2060 due to the continuous fragmentation of legacy P to LMP and LMP to SMP at a rate of 3% per year.

In the GBM-Plastics model, the increasing cumulative MMPW pools of P, LMP, and SMP on land “drive” the amount plastics that are mobilized by runoff to the marine environment (Fig. 2C). The land to sea P, LMP, and SMP summed fluxes toward 2060 therefore show a continuous increase for BAU (43 Tg year^−1^) and Regional (30 Tg year^−1^) scenarios and stabilizing fluxes for Global (22 Tg year^−1^) and SCS (18 Tg year^−1^) scenarios. All of these land to sea plastics fluxes are larger than the 2015 model reference flux of 14 Tg year^−1^. When we follow the increasing land to sea input of MMPW toward 2060, we find that the cumulative amount of floating surface ocean plastic (Fig. 3D) closely tracks land to sea inputs and therefore MMPW policy scenarios. This is a direct consequence of the first-order proportionality of the mass balance equations, where the increase in terrestrial MMPW leads to linear increases in land to sea inputs and surface ocean plastic. Beached P mass keeps growing toward 2060 in BAU and Regional scenarios but stabilizes in Global and SCS scenarios due to declining land to sea transfer and slow but continuous fragmentation of beached P to LMP and SMP. The total amount of marine plastics (P + LMP + SMP, Fig. 3E) increases in all scenarios from 263 Tg in 2015 to 1500 Tg (BAU), 1300 Tg (Regional), 1200 Tg (Global), and 1200 Tg (SCS) in 2060. The primary reason for this continuous increase, despite zero MMPW by 2060 in Global and SCS scenarios, is the large amount of accumulated legacy MMPW on land (Fig. 3B) that continues to be mobilized by runoff to the oceans.

In Fig. 3 and fig. S2, we convert LMP and SMP mass inventories and fluxes to approximate concentrations in key human and wildlife MP exposure environments. Atmospheric boundary layer SMP concentrations of 23 ng m^−3^ in 2015 increase to 100 ng m^−3^ (BAU), 89 ng m^−3^ (Regional), 80 ng m^−3^ (Global), and 74 ng m^−3^ (SCS) by 2060. Indicative SMP concentrations in global river runoff [37,288 km^3^ year^−1^ (*22*)] are estimated at 0.06 mg liter^−1^ in 2015 and increase to 0.28 mg liter^−1^ (BAU), 0.24 mg liter^−1^ (Regional), 0.21 mg liter^−1^ (Global), and 0.19 mg liter^−1^ (SCS) by 2060. SMP concentrations in coastal runoff draining densely populated urban-industrial-agricultural catchments are likely one to two orders of magnitude higher. Indicative surface ocean (upper 50-m mixed layer) SMP concentrations are 6.2 ng liter^−1^ in 2015 and increase to 27 ng liter^−1^ (BAU), 24 (Regional), 21 ng liter^−1^ (Global) and 19 ng liter^−1^ (SCS) by 2060. Surface ocean concentrations in oceanic gyres where plastics accumulate are likely much higher than these global distributed estimates. It is of interest to note that SMP concentrations are 10,000 times higher in continental runoff than in surface ocean waters, suggesting that microplastic exposure in terrestrial aquatic foodwebs is disproportionally larger. Last, SMP concentrations in shelf and slope sediments, as entry point for benthic marine food webs, are 16 mg kg^−1^ in 2015 (on a dry weight basis, in sediments deposited between 1950 and 2015) and reach 140 mg kg^−1^ (BAU), 130 mg kg^−1^ (Regional), 130 mg kg^−1^ (Global), and 120 mg kg^−1^ (SCS) by 2060.

Beyond 2060, the simulations, with plastics production and waste management kept constant at the 2060 values, show important differences driven by MMPW generation and leakage. The BAU scenario leads to unacceptably high amounts and concentrations of plastics in all environments, mostly increasing beyond 2060 due to sustained MMPW generation (15% of total waste). The OECD Regional Action scenario stabilizes plastics concentrations in air and water after 2060 at levels that are three times the 2015 reference values. Because MMPW remains 5% of total waste production in 2060 in the OECD Regional scenario, leakage to the terrestrial, marine, and atmospheric environment remains large. Very different simulation results are reached for OECD Global Action and for the SCS scenarios, where new MMPW reaches ~0% by 2060. This leads to a slow decrease in all leakage pathways, well-illustrated by the peak in 2045, and subsequent decline in land to sea plastic transfer, surface ocean floating plastics mass (Fig. 2, C and D), and water and air MP concentrations (Fig. 3). Ecosystem recovery for LMP is slower than for P, because even if MMPW generation of P after 2060 reaches ~0%, legacy MMPW (for P) continues to fragment on land and supply the terrestrial and marine LMP pools. The recovery for SMP is slower than for LMP for the same reason, and in addition, 0.3% of the mismanaged SMP pool is emitted to air each year, deposited to land and ocean, and re-emitted before eventually settling to the deep ocean and terminal marine sediment sinks. These two factors lead to delayed SMP ecosystem recovery across all Earth surface pools well into the 21st century.

### Plastic and microplastic leakage

The OECD, using its ENV-Linkages model, provides an analysis of the leakage of mismanaged P waste to terrestrial (13 Tg year^−1^) and aquatic (6 Tg year^−1^) environments for the year 2019. An additional 2.7 Tg year^−1^ of MP leakage is estimated, from diverse MMPW and in-use sources, but without explicit fate. Of the aquatic P leakage (6 Tg year^−1^), 1.7 Tg year^−1^ is transported to oceans, where 30 Tg is estimated to have accumulated since 1950 (*4*). The OECD land to sea plastic runoff estimate of 1.7 Tg year^−1^ is a bottom-up estimate based on the global distribution of plastic waste generation derived from population density, gross domestic product per capita, and country scale municipal waste statistics (*23*, *24*). In our GBM-Plastics model, P and MP leakage and land to sea transfer is constrained top-down from the much larger, observed, marine P pool of 114 Tg (IQR: 40 to 197) and MP pool of 149 Tg (IQR: 56 to 207) (Fig. 1E and Table 2). Instead of using population and waste statistics, we used the observed “downstream” marine plastics mass of 263 Tg to calculate what the land to sea transfer must have been. Consequently, our aquatic land to sea P and total plastics transport fluxes of 6.1 and 16 Tg year^−1^ for the year 2019 are significantly larger than the OECD estimate of P (1.7 Tg year^−1^) for 2019. This has important implications for our perception of the magnitude and duration of plastic pollution and exposure associated with the various environmental policy scenarios. First, the increasing number of studies that estimates large amounts of plastics in the deep ocean and marine sediments indicates that plastics and microplastics are four to nine times more mobile than currently assumed. Second, this implies that current and future plastics concentrations, and therefore, human exposure are equally underestimated. Third, the timing of ecosystem recovery is critically dependent on reductions in MMPW generation in the policy scenarios.

Important similarities in ENV-Linkages versus GBM-Plastics models are seen in the relative projections for 2060, illustrated here for the BAU scenario. The OECD projects MMPW generation and leakage to terrestrial and aquatic environments to triple by 2060: Land to sea P transport increases from 1.4 to 4.0 Tg year^−1^, and the marine P pool increases fivefold from 30 to 145 Tg. In Fig. 3, the GBM-Plastics model projects that, under the BAU scenario, land to sea P transport also triples from 6.0 to 17.5 Tg year^−1^, and the marine P pool increases fourfold from 113 to 536 Tg. These similar relative increases in leakage in both ENV-Linkages and GBM-Plastics models reflect the same underlying first-order mass transfer parameterizations: A twofold increase in legacy MMPW on land leads to a twofold increase in leakage from that pool.

### Policy needs

The OECD Global Action and Lau *et al.*’s (*6*) System Change Scenario are ambitious, realistic environmental policy scenarios that aim at reducing the impact of plastic waste on our environment. Here, we ask the question whether they are ambitious enough? In terms of key environmental exposure metrics (Fig. 3), even under Global Action and SCS, the SMP concentrations in air, runoff, ocean water, and shelf sediments in 2060 are only marginally lower than for BAU and are overall 3× higher than in 2019. The payoff of ambitious policy to reach ~0% MMPW comes after 2060 because land to sea dispersal of plastics and emission of SMP depends proportionally on the amount of legacy MMPW on land. Figures 2 and 3 show that OECD Global Action and SCS plastics burdens, fluxes, and exposure decrease systematically after 2045 for P and LMP and after 2075 for SMP, gradually reaching 2019 levels from 2100 onward. If we want ecosystem recovery to be faster, then policy efforts must include active remediation of the terrestrial MMPW pool, transferring recovered plastics to sanitary landfills or incinerating them. We recall, however, that ecosystem recovery also depends on the efficiency of sanitary landfills in retaining plastic and microplastic waste, without further dispersal to ground and surface waters and to air. The landfill P, LMP, and SMP pools are large (Fig. 1), and only minor leakage would offset efforts on MMPW policy. For example, it is estimated that globally there are 100,000 coastal landfills in low-lying areas that are frequently unlined and at risk of erosion, dispersing plastics to the marine environment (*25*).

In summary, the OECD Global Action and Lau *et al.*’s (*6*) System Change scenarios are realistic policy propositions to address the impacts of plastic waste. They have been designed to be practically and economically feasible, and their underlying decrease in virgin plastic production, by substitution and recycling, will also decrease global greenhouse gas emissions from the plastics life cycle. Plastics leakage to the environment and the rapidity of ecosystem recovery have however been underestimated by the OECD. We recommend that Global Action and SCS policy be expanded with remediation of legacy MMPW pools on land and by consolidation and/or remediation of landfilled waste that is at risk of dispersal.

## MATERIALS AND METHODS

### GBM-Plastics v1.1 model

We update the GBM-Plastics v1.0 model (global box model for plastics, updated version 1.1) to simulate how plastic waste is dispersed through the terrestrial, marine, and atmospheric environments upon release or emission (*16*). GBM-Plastics is a first-order box model, meaning that a plastic flux, *F*, out of a reservoir is linearly proportional to the mass, *M*, of plastics in the reservoir, i.e., *F* = *k* × *M*. The mass transfer (rate) coefficients, *k*, are derived for each flux from published observations and estimates of *F* and *M* for the period 2010–2023, from observed degradation or deposition rates, or by fitting. The v1.0 model is described in detail elsewhere, including the 15 mass balance equations for the different reservoirs (*16*). In v1.0, we fitted only 3 of 23 *k* values to illustrate how recent knowledge on *F*, *M*, and *k*, up to 2021 included, could generate a coherent set of *k*’s and a simulated global plastic budget for the year 2015 that agrees within a factor of 10 with observations. In v1.1, several estimates of *F* and *M* have improved (see below) and we decided to fit 12 of 26 *k* values to improve agreement between simulations and observations.

### Marine SMP emission budget update

New observational estimates have been published for marine emission of SMP and for both P and MP in the marine environment, leading to changes in *k* values: In 2022, we assimilated the unique marine SMP emission model estimate of 8.6 (range of 0 to 22) Tg year^−1^ from Brahney *et al.* (*13*), who acknowledged the important uncertainty associated with that estimate. Studies since then indicated the possible overestimation of marine SMP emission, with recent estimates ranging from 0.001 to 8.9 Tg year^−1^ and median SMP emission of 0.11 Tg year^−1^ (IQR: 0.08 to 2.3; Table 1). The large variability depends on the study approach (experimental and modeling), the upper SMP size range that was considered (*26*), and the surface ocean SMP and atmospheric SMP datasets that were used. In this study, we adopted the median SMP marine emission flux of 0.11 Tg year^−1^ and completed it with a similar consensus estimate of land SMP emissions of 0.18 Tg year^−1^ (median, IQR: 0.16 to 0.44; Table 1). We also adopt a revised global atmospheric SMP burden of 0.0036 Tg (median, IQR: 0.002 to 0.017; Table 1) that is based on three studies. This ensemble of SMP burdens and fluxes in the air-sea and air-land system is critical in generating a coherent set of mass transfer coefficients, *k*, for SMP dispersion via atmospheric pathways.

Compared to the preliminary GBM-plastics model and cycle (*16*), the updated model versus 1.1 incorporates 76× lower marine SMP emission and deposition fluxes, associated with a large uncertainty. The simulated lower marine emissions of 0.11 Tg year^−1^ (IQR: 0.08 to 2.32) lead to lower SMP deposition over oceans and land, which in turn decreases the “remote land” SMP pool from 28 Tg previously to 3.3 ± 1.5 Tg. This model estimate needs field-based observations of soil SMP content and/or SMP deposition over remote land areas globally for a closer comparison and model optimization. Note that the mass of SMP in the atmosphere and in marine emission and deposition fluxes is dominated by the upper aerosol size range of 70 μm in the model study results (*15*) that we assimilated. The lower marine SMP emissions lead to approximate lifetimes of SMP in the surface ocean–mixed layer, against emission, of 1.2 years. The lifetime of atmospheric SMP in the planetary boundary layer, against deposition, is 5 days.

### Shelf sediment plastic budget update

We previously estimated the shelf sediment P pool from a review study by Haarr *et al.* (*20*) that approximated mean sea floor P concentrations of 5 Mg km^−2^ (uncertainty not given). Multiplying by the continental shelf surface of 2.89 10^7^ km^2^ resulted in a shelf sediment P pool of 51 Tg (Table 2). We also estimated a shelf sediment MP pool of 65 Tg (1σ, 21 to 78 Tg) from subtidal sediment MP concentrations of 100 MP kg^−1^ (*27*) and a deep sediment MP pool of 1.5 Tg from deep sediment MP concentrations of 0.72 MP g^−1^ (*28*)[see (*16*) for details]. A recent review compiled published microplastic and mesoplastic data in the 10-μm to 25-mm range to estimate a global marine sediment pool of 170 Tg (range of 25 to 900 Tg), dominated by >99% mesoplastic in the 5- to 25-mm range (*18*). Another recent review estimated the global marine sediment macroplastic (P) pool to be 7 Tg (range of 3 to 11 Tg) from remote-operated vehicle studies and 255 Tg (range of 5 to 571 Tg) from bottom trawl studies (*29*). They also estimate that 46% of global marine sediment plastics are deposited to the shelf (<200 m) and 54% to deeper environments. Based on these studies, we derived a best estimate marine sediment P pool of 110 Tg (median, IQR: 39 to 191 Tg) in predominantly shelf, slope, and continental rise environments (Table 2). The total marine plastics (P + LMP + SMP) pools are estimated to be 263 Tg (IQR: 96 to 404).

### Land to sea plastic transport estimate

Estimates of terrestrial plastic inputs to the marine environment remain subject to large variability. Bottom-up estimates based on population and waste management statistics or river plastics concentration and size observations range from 0.1 to 15 Tg year^−1^ (*30*–*33*). Top-down estimates using marine plastic mass balance calculations or three-dimensional marine model plastic inventories can also provide useful estimate of plastics input from land, ranging from 0.5 to 13 Tg year^−1^ (*12*, *16*, *34*), and our box model falls into this category. Top-down model estimates depend directly on the integrated mass of plastics that has accumulated in the marine environment, in sediments, surface and deep ocean waters, and on coastlines, including beaches. The land to sea plastic flux is then adjusted so that integrated historical inputs reproduce the presently (2006–2024) observed mass of plastics in the marine system. Based on our revised marine sediment plastics budget of 263 Tg (Table 2), we adjusted *k* values for land to ocean transfer of P, LMP, and SMP and simulated land to sea transfer fluxes of 6.7, 7.2, and 3.2 Tg year^−1^, respectively (totaling 17 Tg year^−1^) for the year 2019. The direct plastic input of 0.24 T year^−1^ from marine fishing activities (*12*) is implicitly included in our land to sea P flux estimate.

### Surface ocean P, LMP, and SMP budget updates

Surface ocean buoyant P and LMP pools were previously estimated to be on the order of 0.23 and 0.04 Tg (*21*). The inclusion of larger plastic debris in the floating P inventory and the availability of more data have recently produced a 10× larger P estimate of ~2.3 Tg (*17*) and 1.9 Tg (*12*) (Table 2). To model a larger mean surface ocean P pool of 2.1 ± 0.3 Tg, we had to adjust and lower the main outgoing surface ocean P flux in the model, which is sedimentation to the shelf, setting *k* to 38 year^−1^. Because of sampling protocols, surface ocean P and LMP are typically based on neuston net trawling and therefore reflect floating plastics >300 μm. Physical considerations indicate that surface ocean SMP is more rapidly mixed down into the ocean mixed layer (*35*, *36*) and is therefore sampled using in situ pumps or bottles. Table 3 summarizes surface mixed layer SMP observations, extrapolated to the 1- to 300-μm range based on the published particle size distributions. SMP concentrations range from 0.010 to 860 μg m^−3^, with a median value of 8.5 μg m^−3^ (IQR: 1.5 to 44), which multiplied by the global ocean surface of 361,900,000 km^2^ and mean global mixed layer depth of 50 m, yields a surface ocean SMP pool of 0.15 Tg (median, IQR: 0.03 to 0.79).

### Policy scenario details

OECD baseline, Regional Ambition, and Global Ambition policy scenarios for plastics production and waste management from 2019 to 2060 were obtained from (*4*) and aligned with production and waste statistics for 1950 to 2015 by (*3*). We note that this introduces differences in the Fig. 1 budget numbers for the year 2015 compared to the previous version 1.0 of the model (*16*). We also simulate the SCS from Lau *et al.* (*6*) that proposes ambitious but realistic measures to reduce, substitute, recycle, and dispose of plastics. The original SCS scenario provided projections until the year 2040, which we extend here to 2060 by linear extrapolation. SCS plastic production and waste disposal statistics for recent years (2016) are lower than those from (*3*) and (*4*). We therefore anchored (by normalization) the SCS plastic production and waste disposal fractions for the period 2015–2040 to the data for 1950–2015 by Geyer *et al.* (*3*) to maintain intercomparability with OECD scenarios. We acknowledge that our “SCS-like” plastic production and waste disposal estimates deviate to some extent from the original (*6*) estimates, but the overall ambition of the SCS policy trends are preserved. Plastic production and waste management statistics for the four scenarios are summarized in file S1 and include past and projected quantities of plastic waste that is incinerated, recycled, and discarded (landfilled and mismanaged). The model is then run from 1950 to 2100, with only the *k* transfer coefficients and plastics production and waste generation statistics as external forcing. From 2060 to 2100, the model forcings are held constant at the 2060 values. All model uncertainties reported in file S1 are 1σ SD based on 1000 Monte Carlo iterations of model scenario runs. The GBM-Plastics v1.1 model code is included in the Supplementary Materials as Python scripts.

## References

[1] L. Trasande, R. Krithivasan, K. Park, V. Obsekov, M. Belliveau, Chemicals used in plastic materials: An estimate of the attributable disease burden and costs in the United States. J. Endocr. Soc. 8, bvad163 (2024). 38213907 10.1210/jendso/bvad163 PMC10783259

[2] P. J. Landrigan, H. Raps, M. Cropper, C. Bald, M. Brunner, E. M. Canonizado, D. Charles, T. C. Chiles, M. J. Donohue, J. Enck, P. Fenichel, L. E. Fleming, C. Ferrier-Pages, R. Fordham, A. Gozt, C. Griffin, M. E. Hahn, B. Haryanto, R. Hixson, H. Ianelli, B. D. James, P. Kumar, A. Laborde, K. L. Law, K. Martin, J. Mu, Y. Mulders, A. Mustapha, J. Niu, S. Pahl, Y. Park, M.-L. Pedrotti, J. A. Pitt, M. Ruchirawat, B. J. Seewoo, M. Spring, J. J. Stegeman, W. Suk, C. Symeonides, H. Takada, R. C. Thompson, A. Vicini, Z. Wang, E. Whitman, D. Wirth, M. Wolff, A. K. Yousuf, S. Dunlop, The Minderoo-Monaco Commission on plastics and human health. Ann. Glob. Health 89, 23 (2023). 36969097 10.5334/aogh.4056 PMC10038118

[3] R. Geyer, J. R. Jambeck, K. L. Law, Production, use, and fate of all plastics ever made. Sci. Adv. 3, e1700782 (2017). 28776036 10.1126/sciadv.1700782 PMC5517107

[4] OECD, *Global Plastics Outlook: Policy Scenarios to 2060* (Organisation for Economic Co-operation and Development, 2022); www.oecd-ilibrary.org/environment/global-plastics-outlook_aa1edf33-en).

[5] A. Chamas, H. Moon, J. Zheng, Y. Qiu, T. Tabassum, J. H. Jang, M. Abu-Omar, S. L. Scott, S. Suh, Degradation rates of plastics in the environment. ACS Sustainable Chem. Eng. 8, 3494–3511 (2020).

[6] W. W. Y. Lau, Y. Shiran, R. M. Bailey, E. Cook, M. R. Stuchtey, J. Koskella, C. A. Velis, L. Godfrey, J. Boucher, M. B. Murphy, R. C. Thompson, E. Jankowska, A. C. Castillo, T. D. Pilditch, B. Dixon, L. Koerselman, E. Kosior, E. Favoino, J. Gutberlet, S. Baulch, M. E. Atreya, D. Fischer, K. K. He, M. M. Petit, U. R. Sumaila, E. Neil, M. V. Bernhofen, K. Lawrence, J. E. Palardy, Evaluating scenarios toward zero plastic pollution. Science 369, 1455–1461 (2020). 32703909 10.1126/science.aba9475

[7] D. K. A. Barnes, F. Galgani, R. C. Thompson, M. Barlaz, Accumulation and fragmentation of plastic debris in global environments. Philos. Trans. R. Soc. Lond. B Biol. Sci. 364, 1985–1998 (2009). 19528051 10.1098/rstb.2008.0205 PMC2873009

[8] S. Allen, D. Allen, F. Baladima, V. R. Phoenix, J. L. Thomas, G. Le Roux, J. E. Sonke, Evidence of free tropospheric and long-range transport of microplastic at Pic du Midi Observatory. Nat. Commun. 12, 7242 (2021). 34934062 10.1038/s41467-021-27454-7 PMC8692471

[9] X. Peng, M. Chen, S. Chen, S. Dasgupta, H. Xu, K. Ta, M. Du, J. Li, Z. Guo, S. Bai, Microplastics contaminate the deepest part of the world’s ocean. Geochem. Perspect. Lett. 9, doi: 10.7185/geochemlet.1829 (2018).

[10] R. C. Thompson, C. J. Moore, F. S. vom Saal, S. H. Swan, Plastics, the environment and human health: current consensus and future trends. Philos. Trans. R. Soc. Lond. B Biol. Sci. 364, 2153–2166 (2009). 19528062 10.1098/rstb.2009.0053 PMC2873021

[11] L. Lebreton, M. Egger, B. Slat, A global mass budget for positively buoyant macroplastic debris in the ocean. Sci. Rep. 9, 12922 (2019). 31515537 10.1038/s41598-019-49413-5 PMC6742645

[12] M. L. A. Kaandorp, D. Lobelle, C. Kehl, H. A. Dijkstra, E. van Sebille, Global mass of buoyant marine plastics dominated by large long-lived debris. Nat. Geosci. 16, 689–694 (2023).

[13] J. Brahney, N. Mahowald, M. Prank, G. Cornwell, Z. Klimont, H. Matsui, K. A. Prather, Constraining the atmospheric limb of the plastic cycle. Proc. Natl. Acad. Sci. U.S.A. 118, e2020719118 (2021). 33846251 10.1073/pnas.2020719118 PMC8072239

[14] N. Evangeliou, O. Tichý, S. Eckhardt, C. G. Zwaaftink, J. Brahney, Sources and fate of atmospheric microplastics revealed from inverse and dispersion modelling: From global emissions to deposition. J. Hazard. Mater. 432, 128585 (2022). 35299104 10.1016/j.jhazmat.2022.128585

[15] Y. Fu, Q. Pang, S. L. Z. Ga, P. Wu, Y. Wang, M. Mao, Z. Yuan, X. Xu, K. Liu, X. Wang, D. Li, Y. Zhang, Modeling atmospheric microplastic cycle by GEOS-Chem: An optimized estimation by a global dataset suggests likely 50 times lower ocean emissions. One Earth 6, 705–714 (2023).

[16] J. E. Sonke, A. M. Koenig, N. Yakovenko, O. Hagelskjær, H. Margenat, S. V. Hansson, F. De Vleeschouwer, O. Magand, G. Le Roux, J. L. Thomas, A mass budget and box model of global plastics cycling, degradation and dispersal in the land-ocean-atmosphere system. Microplast. Nanoplast. 2, 28 (2022).

[17] M. Eriksen, W. Cowger, L. M. Erdle, S. Coffin, P. Villarrubia-Gómez, C. J. Moore, E. J. Carpenter, R. H. Day, M. Thiel, C. Wilcox, A growing plastic smog, now estimated to be over 170 trillion plastic particles afloat in the world’s oceans—Urgent solutions required. PLOS ONE 18, e0281596 (2023). 36888681 10.1371/journal.pone.0281596 PMC9994742

[18] C. Martin, C. A. Young, L. Valluzzi, C. M. Duarte, Ocean sediments as the global sink for marine micro- and mesoplastics. Limnol. Oceanogr. Lett. 7, 235–243 (2022).

[19] X. Zhu, C. M. Rochman, B. D. Hardesty, C. Wilcox, Plastics in the deep sea—A global estimate of the ocean floor reservoir. Deep Sea Res. 1 Oceanogr. Res. Pap. 206, 104266 (2024).

[20] M. L. Haarr, J. Falk-Andersson, J. Fabres, Global marine litter research 2015–2020: Geographical and methodological trends. Sci. Total Environ. 820, 153162 (2022). 35051476 10.1016/j.scitotenv.2022.153162

[21] M. Eriksen, L. C. M. Lebreton, H. S. Carson, M. Thiel, C. J. Moore, J. C. Borerro, F. Galgani, P. G. Ryan, J. Reisser, Plastic pollution in the world’s oceans: More than 5 trillion plastic pieces weighing over 250,000 tons afloat at sea. PLOS ONE 9, e111913 (2014). 25494041 10.1371/journal.pone.0111913 PMC4262196

[22] A. Dai, K. E. Trenberth, Estimates of freshwater discharge from continents: Latitudinal and seasonal variations. J. Hydrometeorol. 3, 660–687 (2002).

[23] L. Lebreton, A. Andrady, Future scenarios of global plastic waste generation and disposal. Palgrave Commun. 5, 6 (2019).

[24] S. B. Borrelle, J. Ringma, K. L. Law, C. C. Monnahan, L. Lebreton, A. McGivern, E. Murphy, J. Jambeck, G. H. Leonard, M. A. Hilleary, M. Eriksen, H. P. Possingham, H. De Frond, L. R. Gerber, B. Polidoro, A. Tahir, M. Bernard, N. Mallos, M. Barnes, C. M. Rochman, Predicted growth in plastic waste exceeds efforts to mitigate plastic pollution. Science 369, 1515–1518 (2020). 32943526 10.1126/science.aba3656

[25] J. H. Brand, K. L. Spencer, F. T. O’shea, J. E. Lindsay, Potential pollution risks of historic landfills on low-lying coasts and estuaries. WIREs Water 5, e1264 (2018).

[26] D. B. Shaw, Q. Li, J. K. Nunes, L. Deike, Ocean emission of microplastic. PNAS Nexus 2, pgad296 (2023). 37795272 10.1093/pnasnexus/pgad296 PMC10547021

[27] W. J. Shim, S. H. Hong, S. Eo, “Chapter 1 - Marine Microplastics: Abundance, Distribution, and Composition” in *Microplastic Contamination in Aquatic Environments*, E. Y. Zeng, Ed. (Elsevier, 2018), pp. 1–26; www.sciencedirect.com/science/article/pii/B9780128137475000011).

[28] J. Barrett, Z. Chase, J. Zhang, M. M. B. Holl, K. Willis, A. Williams, B. D. Hardesty, C. Wilcox, Microplastic pollution in deep-sea sediments from the Great Australian Bight. Front. Mar. Sci. 7, doi.org/10.3389/fmars.2020.576170 (2020).

[29] X. Zhu, C. M. Rochman, B. D. Hardesty, C. Wilcox, Plastics in the deep sea – A global estimate of the ocean floor reservoir. Deep-Sea Res. I Oceanogr. Res. Pap. 206, 104266 (2024).

[30] J. R. Jambeck, R. Geyer, C. Wilcox, T. R. Siegler, M. Perryman, A. Andrady, R. Narayan, K. L. Law, Plastic waste inputs from land into the ocean. Science 347, 768–771 (2015). 25678662 10.1126/science.1260352

[31] L. C. M. Lebreton, J. van der Zwet, J.-W. Damsteeg, B. Slat, A. Andrady, J. Reisser, River plastic emissions to the world’s oceans. Nat. Commun. 8, 15611 (2017). 28589961 10.1038/ncomms15611 PMC5467230

[32] A. Forrest, L. Giacovazzi, S. Dunlop, J. Reisser, D. Tickler, A. Jamieson, J. J. Meeuwig, Eliminating plastic pollution: How a voluntary contribution from industry will drive the circular plastics economy. Front. Mar. Sci. 6, doi.org/10.3389/fmars.2019.00627 (2019).

[33] L. Mai, X.-F. Sun, L.-L. Xia, L.-J. Bao, L.-Y. Liu, E. Y. Zeng, Global riverine plastic outflows. Environ. Sci. Technol. 54, 10049–10056 (2020). 32700904 10.1021/acs.est.0c02273

[34] Y. Zhang, P. Wu, R. Xu, X. Wang, L. Lei, A. T. Schartup, Y. Peng, Q. Pang, X. Wang, L. Mai, R. Wang, H. Liu, X. Wang, A. Luijendijk, E. Chassignet, X. Xu, H. Shen, S. Zheng, E. Y. Zeng, Plastic waste discharge to the global ocean constrained by seawater observations. Nat. Commun. 14, 1372 (2023). 36914656 10.1038/s41467-023-37108-5 PMC10011382

[35] K. Enders, R. Lenz, C. A. Stedmon, T. G. Nielsen, Abundance, size and polymer composition of marine microplastics ≥10μm in the Atlantic Ocean and their modelled vertical distribution. Mar. Pollut. Bull. 100, 70–81 (2015). 26454631 10.1016/j.marpolbul.2015.09.027

[36] M. Poulain, M. J. Mercier, L. Brach, M. Martignac, C. Routaboul, E. Perez, M. C. Desjean, A. ter Halle, Small microplastics as a main contributor to plastic mass balance in the North Atlantic Subtropical Gyre. Environ. Sci. Technol. 53, 1157–1164 (2019). 30575384 10.1021/acs.est.8b05458

[37] E. van Sebille, C. Wilcox, L. Lebreton, N. Maximenko, B. D. Hardesty, J. A. van Franeker, M. Eriksen, D. Siegel, F. Galgani, K. L. Law, A global inventory of small floating plastic debris. Environ. Res. Lett. 10, 124006 (2015).

[38] S. Yang, T. Zhang, Y. Gan, X. Lu, H. Chen, J. Chen, X. Yang, X. Wang, Constraining microplastic particle emission flux from the ocean. Environ. Sci. Technol. Lett. 9, 513–519 (2022).

[39] Y. Peng, P. Wu, A. T. Schartup, Y. Zhang, Plastic waste release caused by COVID-19 and its fate in the global ocean. Proc. Natl. Acad. Sci. 118, e2111530118 (2021). 34751160 10.1073/pnas.2111530118 PMC8617455

[40] D. Allen, S. Allen, S. Abbasi, A. Baker, M. Bergmann, J. Brahney, T. Butler, R. A. Duce, S. Eckhardt, N. Evangeliou, T. Jickells, M. Kanakidou, P. Kershaw, P. Laj, J. Levermore, D. Li, P. Liss, K. Liu, N. Mahowald, P. Masque, D. Materić, A. G. Mayes, P. McGinnity, I. Osvath, K. A. Prather, J. M. Prospero, L. E. Revell, S. G. Sander, W. J. Shim, J. Slade, A. Stein, O. Tarasova, S. Wright, Microplastics and nanoplastics in the marine-atmosphere environment. Nat. Rev. Earth Environ. 3, 393–405 (2022).

[41] S. Allen, D. Allen, V. R. Phoenix, G. Le Roux, P. Durántez Jiménez, A. Simonneau, S. Binet, D. Galop, Atmospheric transport and deposition of microplastics in a remote mountain catchment. Nat. Geosci. 12, 339–344 (2019).

[42] K. Pabortsava, R. S. Lampitt, High concentrations of plastic hidden beneath the surface of the Atlantic Ocean. Nat. Commun. 11, 4073 (2020). 32811835 10.1038/s41467-020-17932-9 PMC7434887

[43] S. Zhao, E. R. Zettler, R. P. Bos, P. Lin, L. A. Amaral-Zettler, T. J. Mincer, Large quantities of small microplastics permeate the surface ocean to abyssal depths in the South Atlantic Gyre. Glob. Chang. Biol. 28, 2991–3006 (2022). 35048454 10.1111/gcb.16089

[44] S. Eo, S. H. Hong, Y. K. Song, G. M. Han, S. Seo, W. J. Shim, Prevalence of small high-density microplastics in the continental shelf and deep sea waters of East Asia. Water Res. 200, 117238 (2021). 34051457 10.1016/j.watres.2021.117238

[45] P. S. Ross, S. Chastain, E. Vassilenko, A. Etemadifar, S. Zimmermann, S.-A. Quesnel, J. Eert, E. Solomon, S. Patankar, A. M. Posacka, B. Williams, Pervasive distribution of polyester fibres in the Arctic Ocean is driven by Atlantic inputs. Nat. Commun. 12, 106 (2021). 33436597 10.1038/s41467-020-20347-1 PMC7804434

[46] L. D. K. Kanhai, K. Gårdfeldt, O. Lyashevska, M. Hassellöv, R. C. Thompson, I. O’Connor, Microplastics in sub-surface waters of the Arctic Central Basin. Mar. Pollut. Bull. 130, 8–18 (2018). 29866573 10.1016/j.marpolbul.2018.03.011

[47] M. B. Tekman, C. Wekerle, C. Lorenz, S. Primpke, C. Hasemann, G. Gerdts, M. Bergmann, Tying up loose ends of microplastic pollution in the Arctic: Distribution from the sea surface through the water column to deep-sea sediments at the HAUSGARTEN observatory. Environ. Sci. Technol. 54, 4079–4090 (2020). 32142614 10.1021/acs.est.9b06981

