# Supplementary material for: Global environmental plastic dispersal under OECD policy scenarios toward 2060
Source: Sci Adv. 2025 Apr 16;11(16):eadu2396. doi: 10.1126/sciadv.adu2396 (PMC12002131; doi:10.1126/sciadv.adu2396)
Supplement: Supplementary file 1 — Figs. S1 to S3 Table S1 Legends for files S1 and S2 [file sciadv.adu2396_sm.pdf]

Science Advances
AAAS

Supplementary Materials for

Global environmental plastic dispersal under OECD policy scenarios toward 2060

Jeroen E. Sonke *et al.*

Corresponding author: Jeroen E. Sonke, jeroen.sonke@cnrs.fr

Sci. Adv. **11**, eadu2396 (2025)
DOI: 10.1126/sciadv.adu2396

The PDF file includes:

Figs. S1 to S3
Table S1
Legends for files S1 and S2

Other Supplementary Material for this manuscript includes the following:

Files S1 and S2

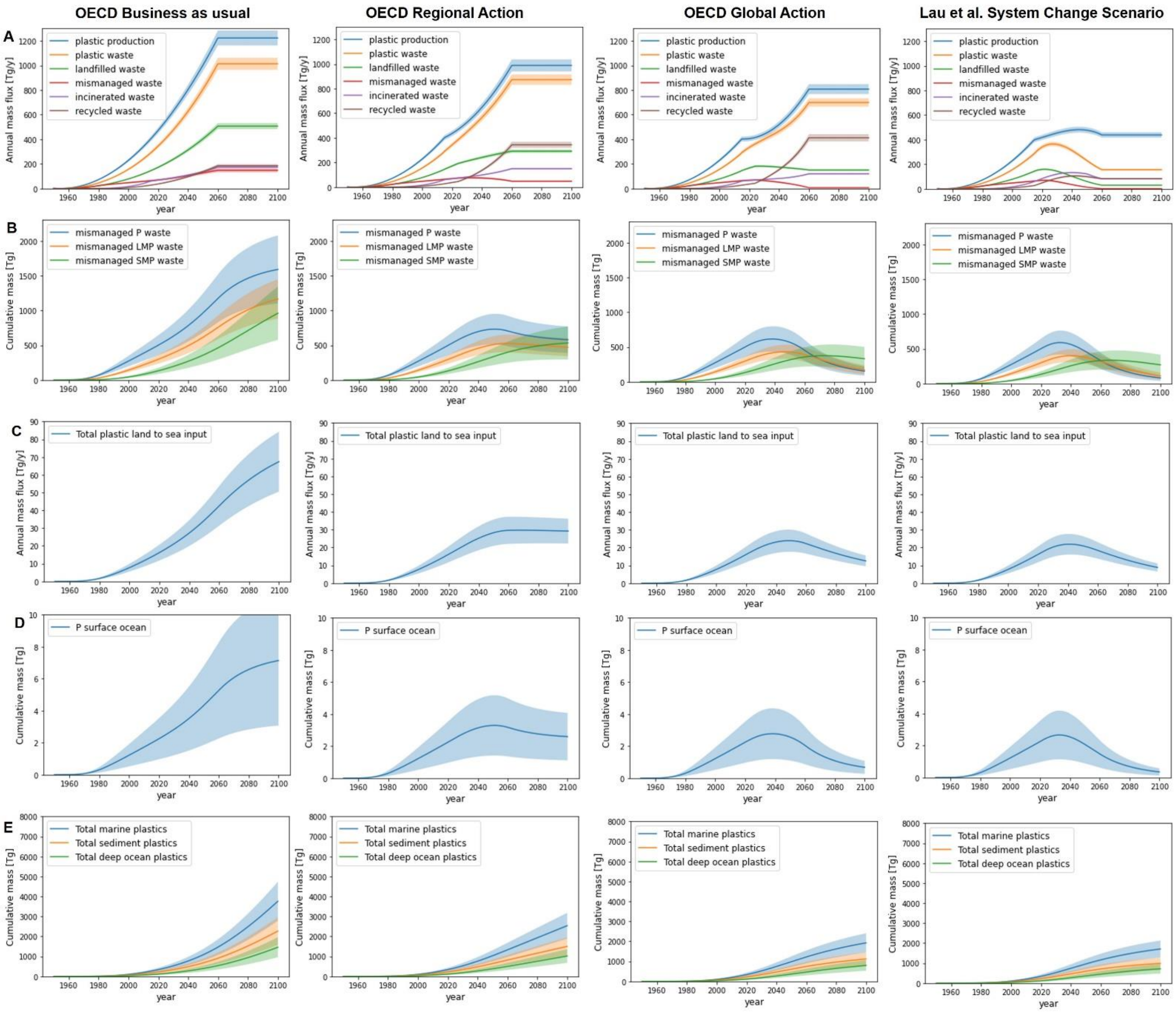

Fig. S1A-E. Model simulations of global plastics dispersal. This figure is similar to Fig. 2 in the main text, but includes the OECD Regional Action scenario for comparison. Four plastics production and waste management scenarios (columns) are simulated from 2016 to 2100: OECD business as usual (BAU), OECD Regional Action, OECD Global Action, and the System Change Scenario (SCS). From 2060 to 2100 the statistics are fixed to the policy scenario endpoint values at 2060. For each scenario the panels show: **(A)** annual plastic production and waste generation; **(B)** cumulative mismanaged plastic waste; **(C)** annual land to sea plastic flux; **(D)** cumulative floating marine macroplastic; **(E)** cumulative plastic mass in the marine system. SMP, small microplastic <300 µm; LMP, large microplastic, 300-5000µm; P, macroplastic >5000µm.

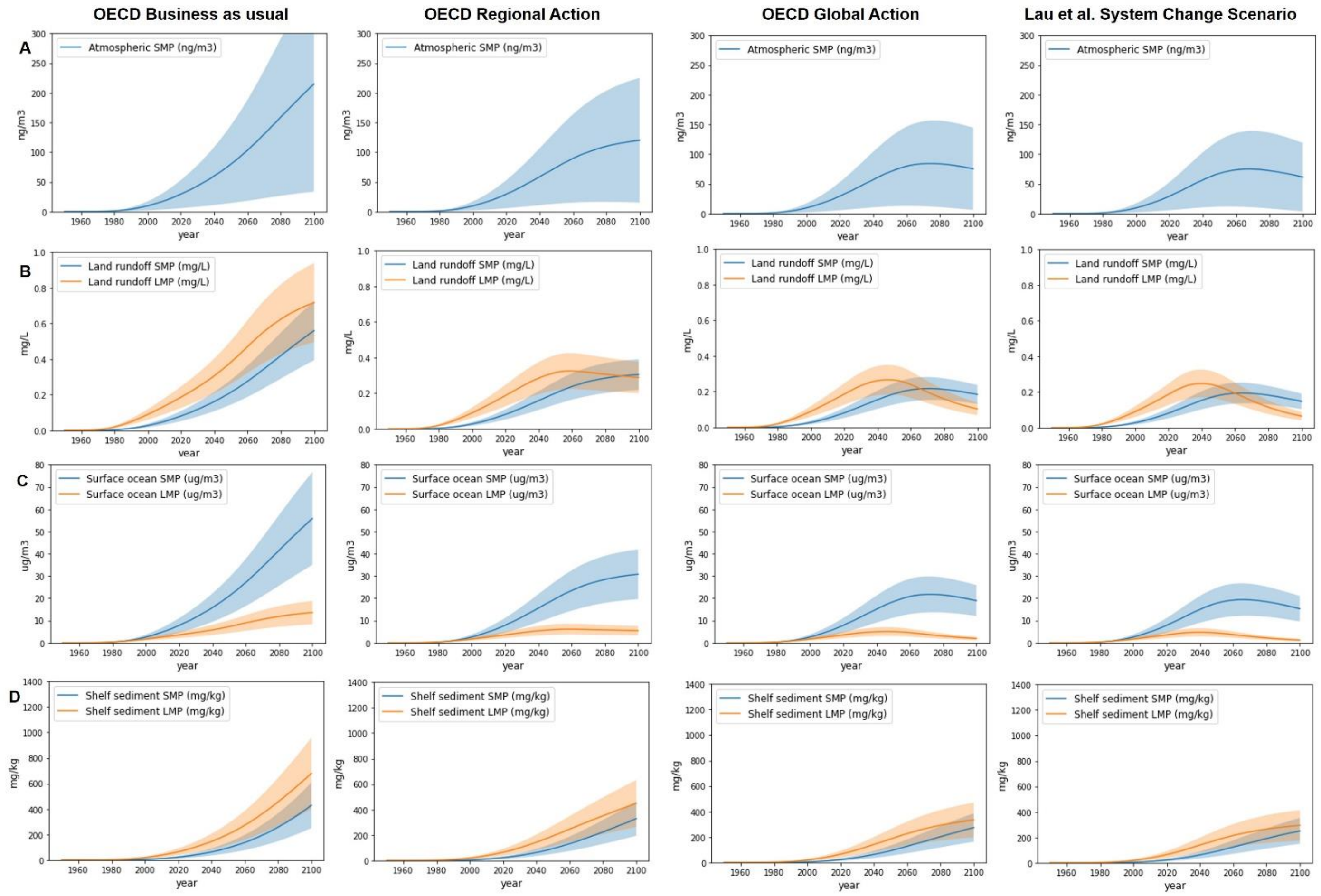

Fig. S2A-D. Model simulations of microplastic concentrations in air, water and sediment. This Figure is similar to Figure 3 in the main text, but includes the OECD Regional Action scenario for comparison. GBM-Plastics simulations of four plastics production and waste management scenarios (columns) from 2016 to 2100: OECD business as usual (BAU), OECD Regional Action, OECD Global Action, and the System Change Scenario (SCS) from (6). For each scenario the panels show microplastic concentrations in: **(A)** the atmosphere (troposphere); **(B)** land runoff to oceans; **(C)** surface ocean mixed layer (50m); **(D)** continental shelf sediment. SMP, small microplastic <300 µm; LMP, large microplastic, 300-5000µm.

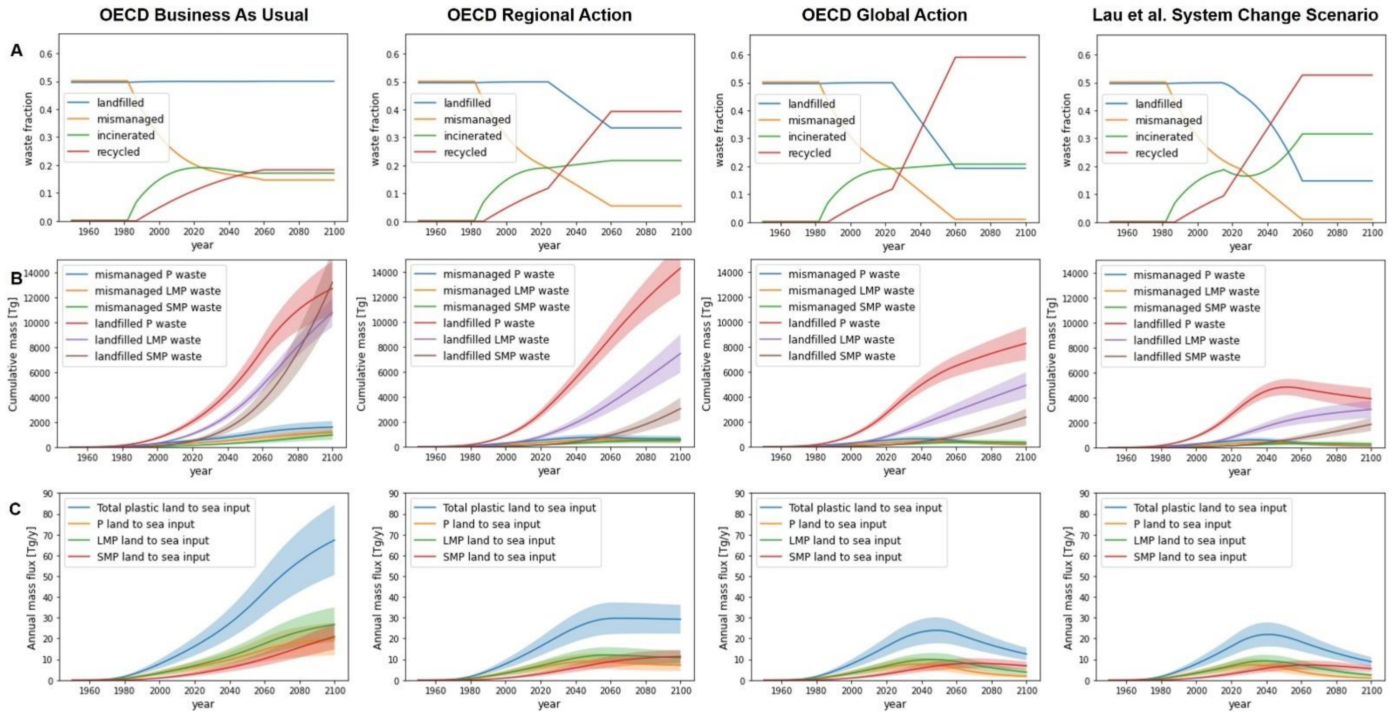

Fig. S3A-C. Summary of mismanaged and landfilled plastic waste and its transport to sea. GBM-Plastics simulations of four plastics production and waste management scenarios (columns) from 2016 to 2100: OECD business as usual (BAU), OECD Regional Action OECD Global Action, and the System Change Scenario (SCS) from (6). For each scenario the panels show: (A) waste management projections; (B) comparative managed (landfilled) and mismanaged plastic waste; (C) land to sea plastic inputs. SMP, small microplastic <300 µm; LMP, large microplastic, 300-5000µm; P, macroplastic >5000µm.

Table S1. Summary of observed and modeled plastic pools and fluxes. The pool size and fluxes complements Fig. 1 for the global plastic cycling budget for the year 2015, and for the four OECD and SCS policy scenarios for the year 2060. BAU, business as usual; Regonal, OECD regional action; Global, OECD global ambition; SCS, system change scenario; sd, standard deviation. SMP, small microplastic <300 µm; LMP, large microplastic, 300-5000µm; P, macroplastic >5000µm. MP indicates the sum of LMP and SMP.

**Parameter**	**Units**		**Observed**	**Modeled**	**Modeled**	**Modeled**	**Modeled**	**Modeled**
				**BAU**	**BAU**	**Regional**	**Global**	**SCS**
**Year**			**2015**	**2015**	**2060**	**2060**	**2060**	**2060**
In-use P+LMP	Tg	mean		2599	10157	6686	5672	9877
	Tg	sd		488	2681	2366	2057	1697
P waste in landfill	Tg	mean		2073	11271	8713	6470	4719
	Tg	sd		259	1315	1051	839	687
LMP waste in landfill	Tg	mean		603	4155	3482	2858	2371
	Tg	sd		121	808	690	584	505
SMP waste in landfill	Tg	mean		83	973	902	827	768
	Tg	sd		27	300	277	254	236
P waste to CO2	Tg	mean		1421	8090	7666	6424	4660
	Tg	sd		463	2055	1663	1380	1105
P floating at surface ocean	Tg	mean	1.9	2.0	5.3	3.2	2.0	1.5
	Tg	sd		1.1	3.0	1.8	1.2	0.9
LMP floating at surface ocean	Tg	mean	0.04	0.06	0.16	0.11	0.08	0.07
	Tg	sd		0.02	0.06	0.04	0.03	0.03
SMP floating at surface ocean	Tg	mean	0.15	0.11	0.49	0.42	0.38	0.35
	Tg	sd		0.05	0.19	0.16	0.14	0.13
MP suspended in deep ocean	Tg	mean	82	87	541	499	465	446
	Tg	sd		35	207	191	178	171
SMP in atmosphere	Tg	mean	0.0036	0.005	0.022	0.019	0.017	0.016
	Tg	sd		0.004	0.018	0.016	0.014	0.013
SMP in remote land surfaces	Tg	mean		2.7	19	18	17	17
	Tg	sd		1.7	13	12	11	11
P on sandy beaches	Tg	mean	1.3	1.5	5.4	4.4	3.7	3.3
	Tg	sd		1.4	5.1	4.2	3.6	3.2
MP on sandy beaches	Tg	mean	0.5	0.7	5.1	4.8	4.6	4.4
	Tg	sd		0.7	4.7	4.4	4.2	4.1
P deposited to shelf+slope sediments	Tg	mean	110	110	525	460	413	386
	Tg	sd		45	211	184	165	154
MP deposited to shelf+slope sediments	Tg	mean	65	60	376	347	324	311
	Tg	sd		26	159	146	136	131
MP deposited to deep sediments	Tg	mean	1.0	1.0	9.3	8.9	8.6	8.5
	Tg	sd		0.5	4.3	4.1	4.0	3.9
P in MMPW pool	Tg	mean		442	1176	703	452	328
	Tg	sd		126	345	217	150	117
LMP in MMPW pool	Tg	mean		257	759	526	390	317
	Tg	sd		53	169	127	103	89
SMP in MMPW pool	Tg	mean		106	464	402	357	330
	Tg	sd		35	169	151	138	131
P and LMP production flux (incl reycling)	Tg/y	mean		402	1223	989	807	438
	Tg/y	sd		20	61	49	40	22
discarded P waste flux to MMPW	Tg/y	mean		55	128	41	6	1
	Tg/y	sd		6	15	5	0.7	0.2
discarded LMP waste flux to MMPW	Tg/y	mean		8.8	20	7	1.0	0.2
	Tg/y	sd		2.1	4.9	1.6	0.2	0.1
discarded P waste flux to landfill	Tg/y	mean		124	436	251	130	26
	Tg/y	sd		10	34	20	10	2.0
discarded LMP waste flux to landfill	Tg/y	mean		20	70	40	21	4.1
	Tg/y	sd		5	16	9	5	0.9
incinerated waste flux	Tg/y	mean		60	174	150	120	82
	Tg/y	sd		3	8	7	6	4
open burning of P flux	Tg/y	mean		25	57	19	2.7	0.6
	Tg/y	sd		8	20	6	0.9	0.2
P waste recycled flux	Tg/y	mean		27	185	343	412	82
	Tg/y	sd		2	13	24	29	6
P fragmentation to LMP in MMPW pool	Tg/y	mean		13	35	21	13	10
	Tg/y	sd		4	11	6	4	3
LMP fragmentation to SMP in MMPW pool	Tg/y	mean		8	23	16	12	9
	Tg/y	sd		2	7	5	4	3
P fragmentation to LMP in landfill pool	Tg/y	mean		20	110	84	62	45
	Tg/y	sd		6	29	21	15	11
LMP fragmentation to SMP in landfill pool	Tg/y	mean		6.0	41	34	28	23
	Tg/y	sd		1.9	12	10	8	7
land to sea P flux	Tg/y	mean		5.6	14.7	8.7	5.6	4.0
	Tg/y	sd		2.2	5.9	3.5	2.2	1.6

Parameter	Units		Observed	Modeled	Modeled	Modeled	Modeled	Modeled
				BAU	BAU	Regional	Global	SCS
Year			2015	2015	2060	2060	2060	2060
land to sea LMP flux	Tg/y	mean		6.0	17.5	12.1	8.9	7.2
	Tg/y	sd		1.9	5.5	3.8	2.8	2.2
land to sea SMP flux	Tg/y	mean		2.4	10.3	8.8	7.8	7.2
	Tg/y	sd		0.9	3.3	2.8	2.4	2.2
land to sea P+LMP+SMP flux	Tg/y	mean	0.006 - 15	13.9	43	30	22	18
	Tg/y	sd		3.9	11	8	6	5
surface ocean P beaching flux	Tg/y	mean		0.10	0.28	0.17	0.11	0.08
	Tg/y	sd		0.10	0.25	0.15	0.10	0.07
surface ocean P sedimentation to shelf+slope	Tg/y	mean		5.3	14.2	8.5	5.5	4.0
	Tg/y	sd		2.2	5.7	3.4	2.2	1.6
P fragmentation to LMP in surface ocean	Tg/y	mean		0.06	0.15	0.09	0.06	0.04
	Tg/y	sd		0.03	0.09	0.05	0.03	0.02
surface ocean LMP beaching flux	Tg/y	mean		0.003	0.009	0.006	0.004	0.004
	Tg/y	sd		0.003	0.007	0.005	0.004	0.003
surface ocean LMP sedimentation to shelf+slope	Tg/y	mean		2.4	7.2	4.9	3.6	2.9
	Tg/y	sd		1.0	3.0	2.0	1.5	1.2
surface ocean LMP settling to deep ocean	Tg/y	mean		3.6	10.5	7.2	5.3	4.3
	Tg/y	sd		1.4	3.9	2.6	1.9	1.6
LMP fragmentation to SMP in surface ocean	Tg/y	mean		0.002	0.005	0.003	0.002	0.002
	Tg/y	sd		0.001	0.002	0.001	0.001	0.001
surface ocean SMP sedimentation to shelf+slope	Tg/y	mean		1.0	4.4	3.8	3.4	3.1
	Tg/y	sd		0.5	1.8	1.6	1.4	1.3
surface ocean SMP settling to deep ocean	Tg/y	mean		1.5	6.4	5.5	4.9	4.5
	Tg/y	sd		0.6	2.3	2.0	1.7	1.6
surface ocean SMP emission to atmosphere	Tg/y	mean	0.11	0.15	0.65	0.56	0.50	0.46
	Tg/y	sd		0.21	0.92	0.79	0.70	0.65
deep ocean LMP sedimentation	Tg/y	mean		0.06	0.25	0.21	0.19	0.17
	Tg/y	sd		0.03	0.11	0.10	0.08	0.08
deep ocean SMP sedimentation	Tg/y	mean		0.01	0.09	0.09	0.08	0.08
	Tg/y	sd		0.01	0.04	0.04	0.04	0.04
atmospheric SMP deposition to remote land	Tg/y	mean		0.25	1.09	0.95	0.84	0.78
	Tg/y	sd		0.15	0.70	0.61	0.55	0.51
atmospheric SMP deposition to surface ocean	Tg/y	mean		0.16	0.73	0.63	0.56	0.52
	Tg/y	sd		0.20	0.89	0.77	0.69	0.64
remote land SMP emission to atmosphere	Tg/y	mean		0.01	0.06	0.06	0.05	0.05
	Tg/y	sd		0.01	0.09	0.08	0.08	0.08
remote land SMP runoff to surface ocean	Tg/y	mean		0.06	0.43	0.40	0.38	0.37
	Tg/y	sd		0.04	0.26	0.24	0.23	0.22
beach P fragmentation to LMP	Tg/y	mean		0.04	0.16	0.13	0.11	0.09
	Tg/y	sd		0.04	0.14	0.12	0.10	0.09

Captions for additional supplementary files

File S1 Model forcings and simulation output data for four policy scenarios. This Microsoft Excel file contains six worksheets: sheet 1. Summary of model forcings, including plastics production and waste generation statistics from 1950 to 2060, for each of the four policy scenarios; sheets 2-5. Comprehensive model output for the four policy scenarios; sheet 6. Summary of model output for the four policy scenarios.

File S2 Python model source code used to evaluate the OECD policy scenarios. The GBM-Plastics-v1.1 python model code consists of a Read Me file, a model forcings input file, and 4 python scripts needed to execute the model and plot the figures for this study.
